# Potential Gene Association Studies of Chemotherapy-Induced Cardiotoxicity: A Systematic Review and Meta-Analysis

**DOI:** 10.3389/fcvm.2021.651269

**Published:** 2021-06-04

**Authors:** Xinyu Yang, Guoping Li, Manke Guan, Aneesh Bapat, Qianqian Dai, Changming Zhong, Tao Yang, Changyong Luo, Na An, Wenjing Liu, Fan Yang, Haie Pan, Pengqian Wang, Yonghong Gao, Ye Gong, Saumya Das, Hongcai Shang, Yanwei Xing

**Affiliations:** ^1^Key Laboratory of Chinese Internal Medicine of the Ministry of Education, Dongzhimen Hospital Affiliated to Beijing University of Chinese Medicine, Beijing, China; ^2^Guang'anmen Hospital, China Academy of Chinese Medical Sciences, Beijing, China; ^3^Cardiovascular Research Center, Massachusetts General Hospital and Harvard Medical School, Boston, MA, United States; ^4^College of Traditional Chinese Medicine, Beijing University of Chinese Medicine, Beijing, China; ^5^Institute of Chinese Materia Medica, China Academy of Chinese Medical Sciences, Beijing, China; ^6^Department of Critical Care Medicine, Shanghai Medical College, Huashan Hospital, Fudan University, Shanghai, China

**Keywords:** chemotherapy, cardiotoxicity, gene, SNPs, meta-analysis

## Abstract

Chemotherapy is widely used in the treatment of cancer patients, but the cardiotoxicity induced by chemotherapy is still a major concern to most clinicians. Currently, genetic methods have been used to detect patients with high risk of chemotherapy-induced cardiotoxicity (CIC), and our study evaluated the correlation between genomic variants and CIC. The systematic literature search was performed in the PubMed, Cochrane Central Register of Controlled Trials (CENTRAL), China Biology Medicine disc (CBMdisc), the Embase database, China National Knowledge Internet (CNKI) and Wanfang database from inception until June 2020. Forty-one studies were identified that examined the relationship between genetic variations and CIC. And these studies examined 88 different genes and 154 single nucleotide polymorphisms (SNPs). Our study indicated 6 variants obviously associated with the increased risk for CIC, including CYBA rs4673 (pooled odds ratio, 1.93; 95% CI, 1.13–3.30), RAC2 rs13058338 (2.05; 1.11–3.78), CYP3A5 rs776746 (2.15; 1.00–4.62) ABCC1 rs45511401 (1.46; 1.05–2.01), ABCC2 rs8187710 (2.19; 1.38–3.48), and HER2-Ile655Val rs1136201 (2.48; 1.53–4.02). Although further studies are required to validate the diagnostic and prognostic roles of these 6 variants in predicting CIC, our study emphasizes the promising benefits of pharmacogenomic screening before chemotherapy to minimize the CIC.

## Introduction

The burgeoning field of cardio-oncology is continuing to grow in step with major scientific developments in oncology that have improved cancer prognosis and survivorship (1). Cardiotoxicity has long been considered one of the main side effects of chemotherapy in cancer patients (2–6). Now, more effective therapies and some forms of radiotherapy may also have multiple cardiovascular (CV) secondary effects, in particularly left ventricular dysfunction (LVD) and heart failure (HF) (7–12). Some of the data from studies on the genetic defects and pharmacological interventions suggested that many molecules, primarily those regulating oxidative stress (OS), autophagy, apoptosis, and metabolism, contributing to the pathogenesis of cardiotoxicity induced by cancer treatment. Treatment with anthracyclines has been reported to increase the risk of cardiotoxicity and death by more than five times (13–15). Early diagnosis and treatment of cardiotoxicity can increase the chances of recovery (16, 17), which highlights the urgent need to develop new technologies and programmes for the early management of cardiotoxicity caused by chemotherapy and for a multidisciplinary clinical approach throughout the chemotherapy process (18–20).

Early identification of the CIC is essential to minimize the harmful side effects of cancer treatment, and could provide oncologists and cardiologists with an ideal choice to allow personalized antitumor treatment strategies or interventions (21). Although some factors may make some patients more susceptible to the severity of toxicity, individual differences in toxicity manifestations still remain large, which will significantly exacerbate these toxic. Genetics, therefore, could provide insights into the development of toxicity induced by some cancer treatments. Identification of the genetic biomarkers that are able to predict whether the patient is at risk of developing cancer therapies-induced cardiac dysfunction will allow for the minimization of cardiotoxicities during cancer treatment by careful monitoring, applying cardioprotective drugs or using optimized cancer therapies. Several recent studies have showed the role of genetic variation as a biomarker for the early detection of CIC (22–25). The aim of this study was to provide an overview of studies focusing on the relationship between polymorphic gene variants and CIC.

## Methods

### Search Strategy

Our study searched the PubMed, CENTRAL, CBMdisc, the Embase database, Wanfang database and CNKI from the beginning to June 2020. The search terms include chemotherapy, anthracyclines, doxorubicin, daunorubicin, epirubicin, idarubicin, trastuzumab, cyclophosphamide, 5-fluorouracil, methotrexate, adriamycin, cisplatin, cytoxan, cardiotoxicity, HF, cardiomyopathy, arrhythmia, genetic, pharmacogenomics, variant and polymorphism. The search is limited to clinical trials involving human participants. Then, literature titles, abstracts and subject words are carefully analyzed to further identify keywords for document retrieval. If the abstract is relevant to our research, we will read the full text. References in the research were also analyzed to find out some studies that might have been missed in the original search.

### Study Selection

Studies that met the criteria were as follows: (i) most of the SNPs were considered as dominant inheritance models unless specifically notified; (ii) original studies that determine the relationship between the genetic polymorphism (including different SNPs in each gene deletions, duplication, and copy-number variants) and cardiotoxicity; (iii) chemotherapy was used regardless of the cycle regimen type, timing, and duration of administration; (iv) in human studies and (vi) in English language. Exclusion criteria: (i) laboratory studies, case series and reports, interim studies; (ii) republication literature; (iii) data with obvious error. The relevance of the article titles and abstract was filtered by two independent reviewers, and the full text was retrieved based on inclusion criteria. Any disagreement will be settled by the third author by decision.

### Data Extraction

The authors (GPL and MKG) extracted the data and checked the qualifications and the methodological quality of each included study. Any disagreements will be discussed and if the discussion is not finalized, the disagreements would be resolved by the third author (XYY). This information extracted from each document included the name of the first author, the year of publication, the sample size of the trial, the type of participant, the age of the participant, the type of cancer, the genotyping technique and the definition of cardiotoxicity. At last, we assessed the relationship between different genomic polymorphisms and cardiotoxicity.

### Quality Assessment

The STREGA reporting guide was used to assess the quality of each study report (26, 27). STREGA includes five main categories of information: reporting possible genotyping methods and errors, addressing population stratification methods, methods used to inference haplotypes or genotypes, whether the Hardy-Weinberg equilibrium was considered and whether this study is the first to report genetic associations, replication work or both. The quality of the report is assessed independently by two investigators, and differences are resolved through the discussion or through the third author if no consensus is reached between the two investigators.

### Statistical Analysis

The meta-analysis was analyzed using the Review Manager 5.3 packages (http://comunity.cochrane.org/tools/review-production-tools/revman-5) (28) and STATA version 13.0. In studies evaluating the same genotype polymorphism, we performed the meta-analysis using the fixed effects (FE) model and the random effects model (29), and this study heterogeneity was evaluated using the I^2^ statistics. Overall heterogeneity was quantified with the I^2^, with *p* < 0.01 used to indicate significance (30). Compared with the sampling error in the study, the true variance ratio of the estimated effects between the included studies was calculated by using the I^2^ statistic and moderate heterogeneity is considered when I^2^ is between 50 and 75% and high heterogeneity is considered when I^2^>75% (30). The sensitivity analysis was conducted to evaluate the stability of this study, namely meta-analysis is performed again after the exclusion of abnormal results, and the results of meta-analysis were compared with those of studies that did not exclude abnormal results.

## Result

### Study and Patient Characteristics

**Figure 1:**
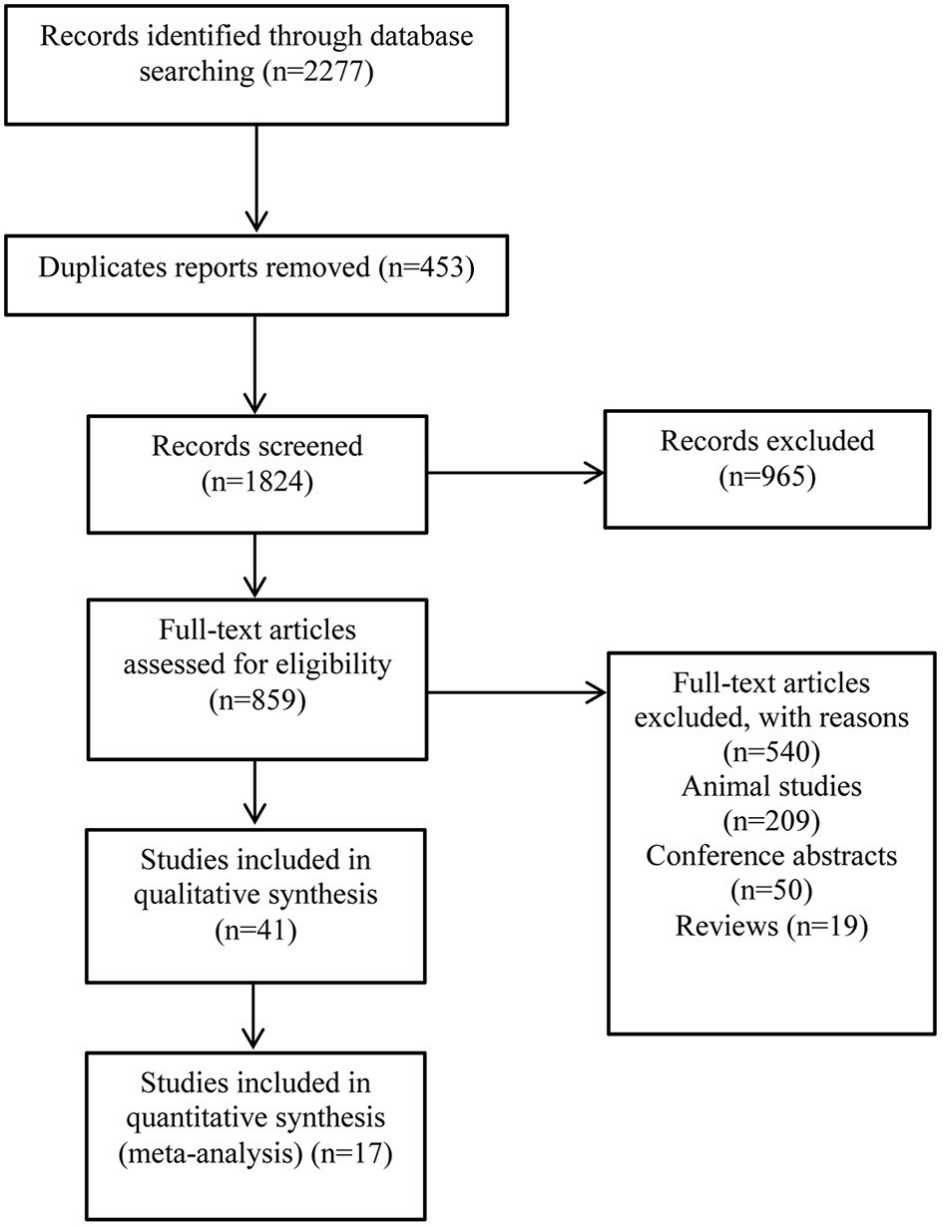
Selection process and criteria for inclusion in this study.

The study searched 2,277 literatures and 859 that were evaluated. A total of forty-one studies involving in 9,183 patients, were included in this study (Figure 1). Table 1 lists the characteristics of the included studies. Thirteen studies were case-control studies (22, 23, 32, 35, 36, 39, 44, 51, 53, 54, 60, 63, 64), eight were nested case-control studies (24, 25, 31, 34, 37, 48, 52, 59). Another eleven were prospective cohort studies (33, 41–43, 45, 46, 50, 55–57, 62) while eight were retrospective cohort study (38, 40, 48, 58, 61, 65–67). The remaining one was a case report (49). An almost equal number of studies have been performed in children (*n* = 15) and adults (*n* = 19). Seven studies included children and adults in our study. The most common cancer types examined include breast cancer (BC) (*n* = 12), leukemia (*n* = 9), osteosarcoma (*n* = 1), lymphoma (*n* = 3), and hematological neoplasms (*n* = 3). In eleven other studies, mixed cancers were examined.

**Table 1:** Genetic polymorphisms in chemotherapy-induced cardiotoxicity (CIC).

**Study**	**Participants**	**Gender: male/female**	**Age(years): case/control**	**Drug used**	**Type of Cancer**	**Source of DNA sample**	**Genotyping**	**Cardiac toxicity**
Wojnowski et al. (31)	NCC; 550	50/37; 212/151	62.0 ± 10.9; 61.3 ± 11.0	Doxorubicin	Non-Hodgkin lymphoma	Peripheral blood	Pyrosequencing; PCR	Arrhythmia, CHF, myocarditis-pericarditis
Weiss et al. (32)	CC; 197	~98/99	68 (56–88)	Daunorubicin	AML	Peripheral blood	Multiplex PCR	SWOG toxicity
Beauclair et al. (33)	PC; 61	NR	50.7 (30.5–83.1)	Trastuzumab	BC	Blood	PCR	Decreased LVEF
Blanco et al. (34)	NCC; 145	10/20; 57/58	10.3 ± 6.5; 9.1 ± 5.8	Anthracyclines	Childhood cancer	Buccal cells/saliva	PCR-RFLP; allelic discrimination with specific fluorescent probes	CHF
Rossi et al. (35)	CC; 106	55/51; 55/51	66 (56–75)	Doxorubicin	Large B-cell lymphoma	Peripheral blood	SNP minisequencing	Abnormalities ECG
Rajićet al. (36)	CC; 76	32/44	25.8 ± 5.3	Anthracyclines	ALL	Bone marrow smears	qPCR; TaqMan genotyping assay	Cardiac damage, SF <30%, LVEF <54%
Blanco et al. (37)	NCC; 487	76/94; 162/155	8.3 ± 6; 8.2 ± 6	Anthracyclines	Childhood cancer	Peripheral blood/buccal cells/saliva	Allelic discrimination with specific fluorescent probes	Cardiomyopathy, EF <40%, SF <28%
Semsei et al. (38)	RC; 235	126/109	5.7 ± 3.8	Anthracyclines	ALL	Peripheral blood	Minisequencing; Genome Lab SNP stream genotyping assay	LV dysfunction; reduced LVFS
Visscher et al. (39)	CC; 440	17/21; 66/52	5.5 (0.04–17.0); 3.9 (0.5–16.5)	Anthracyclines	Childhood cancer	NR	Custom Illumina GoldenGate SNP genotyping assay	CHF; SF <26%
Cascales et al. (40)	CR; 97	37/12; 28/20	60 ± 12; 44 ± 18	Anthracyclines	Hematological neoplasms	Blood	PCR	HF; LVEF decrease; EF <50%
Volkan-Salanci et al. (41)	PC; 70	7/63	49.1 ± 13.6	Anthracyclines	BC	NR	TaqMan genotyping assay	Cardiac dysfunction; LVEFs <50%
Lubieniecka et al. (42)	PC; 185	86/99	46 (14–74)	Anthracyclines	AML	Blood	Sequenom genotyping assay	LVEF % drop
Kitagawa et al. (43)	PC; 34	0/34	49 (21–71)	Epirubicin, cyclophosphamide, 5-fluorouracil	BC	Whole blood	TaqMan genotyping assay	Arrhythmias; QTc interval prolongation
Windsor et al. (44)	CC; 58	34/24	18 (10–51)	MAP	Osteosarcoma	Peripheral blood	PCR; Illumina microarray	Decreased LVEF
Roca et al. (45)	PC; 392	NR	48 (24–65)	5-fluorouracil, epirubicin, cyclophosphamide	BC	Whole blood	PCR	CHF; LVEF <50%
Lipshultz et al. (46)	PC; 184	101/83	15.2 (3.1–31.4)	Doxorubicin	ALL	Peripheral blood	Pyrosequencing; Sequenom genotyping assay; TaqMan genotyping assay	Cardiac dysfunction; LVEF, cTnT, NT-proBNP
Armenian et al. (47)	NCC; 255	34/43; 119/59	49.2 (16–68.8); 51.0 (6.4–72.6)	Anthracyclines	Hematological neoplasms	Peripheral blood	Sequenom MassARRAY	Sign and symptoms
Lubieniecka (48)	RC; 91	48/43	48.4 (19–74)	Daunorubicin	AML	Blood	Sequenom genotyping assay	Decreased LVEF
Sachidanandam et al. (49)	CR; 2	0/2	NR	Doxorubicin	Childhood cancer	Whole blood	PCR	Sign and symptoms
Vivenza et al. (50)	PC; 48	1/47	57.5 (28–73)	Anthracyclines	BC	Blood	Allelic discrimination; TaqMan genotyping assay	Decreased LVEF; LVEF <50%
Visscher et al. (51)	CC; 218	31/25; 75/87	21.7 (1.4–33.8); 16.1 (2.3–33.7)	Anthracyclines	Childhood cancer	Blood/saliva/buccal swab	Custom Illumina GoldenGate SNP genotyping assay	SF <24% or symptoms, CTCAE grade 2–4
Wang et al. (52)	NCC; 363	40/53; 94/100	19.4 (0.4–41.7); 18.5 (3.5–49.2)	Anthracyclines	Children's Oncology	Peripheral blood, buccal cells/ saliva	Illumina IBC cardiovascular SNP array	American Heart Association criteria
Wasielewski et al. (53)	CC; 21	NR	49 (2–57)	Anthracyclines	NR	NR	Targeted next-generation DNA sequencing	Signs and symptoms; cardiomyopathy
Krajinovic et al. (54)	CC; 295	134/117; 21/23	6.16; 5.27	Doxorubicin	ALL	Blood, buccal swabs	PCR	Reduction of EF and FS
Visscher et al. (23)	CC; 536	64/58; 211/187	7.4 (0.04–17.6); 4.9 (0.1–17.7)	Anthracyclines	Childhood cancer	Blood, saliva, buccal swabs	Illumina GoldenGate SNP genotyping assay	FS ≤ 26%, LV dysfunction
Peña et al. (55)	PC; 78	NR	51.72	Trastuzumab	BC	Saliva	TaqMan allelic discrimination assay	CHF, LVEF <50%
Aminkeng et al. (56)	PC; 376	27/27; 174/148	16.5 (7.5–26); 15 (8–21.5)	Anthracyclines	Pediatric oncology	NR	Illumina HumanOmniExpress assay	FS ≤ 24%, LVEF <45%
Reichwagen et al. (24)	NCC; 520	25/31; 46/48	68 (61–80); 67 (62–79)	Doxorubicin	CD20^+^ B-cell lymphomas	Blood	Pyrosequencing; TaqMan genotyping assays	Arrhythmia, reduced EF
Vulsteke et al. (57)	PC; 877	NR	50.3	Epirubicin	BC	Blood	Sequenom MassARRAY	Decrease LVEF, LVEF >10%
Stanton et al. (58)	RC; 140	0/140	56 (32–85)	Trastuzumab	BC	Peripheral blood	PCR	LVEF <55%
Hertz et al. (22)	CC; 166	0/19; 0/147	50 (35–64); 50 (24–80)	Doxorubicin	BC	Blood	Sequenom MassARRAY; TaqMan allelic discrimination assay	EF <55%
Reinbolt et al. (59)	NCC; 162	0/52; 0/110	51.9 ± 11.9; 50.1 ± 9.3	Adriamycin, and cytoxan	BC	NR	TaqMan allelic discrimination assay	EF <50%
Wang et al. (25)	NCC; 385	76/90; 106/113	16.1 ± 10.7; 8.3 ± 5.8	Anthracyclines	Childhood cancer	Blood, buccal cells, saliva	Illumina HumanOmniExpress assay; Sequenom MassARRAY	Cardiomyopathy, EF <40%, SF <28%
Schneider et al. (60)	CC; 102	NR	NR	Anthracyclines	BC	NR	Illumina Genotyping	CHF; LVEF <50%
Ruiz-Pinto et al. (61)	RC; 154	0/71; 53/30	54.3; 7.8	Anthracyclines	BC	NR	Illumina HumanExome BeadChip array	Cardiac failure, LVEF decreased
Pop-Moldovan et al. (62)	PC; 25	13/12	59.6	Doxorubicin	Hematological neoplasms	Blood	qRT-PCR	Diastolic dysfunction; LVEF decreased
Ruiz-Pinto et al. (63)	CC; 93	33/25; 25/10	5.1; 10.4	Anthracyclines	Pediatric cancer	Peripheral blood	Illumina HumanExome BeadChip array	LV dysfunction
Huang et al. (64)	CC; 36	22/14	7.1 ± 2.3	Daunorubicin	ALL	Bone marrow	PCR	Abnormal ECG
Sági et al. (65)	RC; 680	NR	6.6 (± 4.3)	Anthracyclines	ALL	Peripheral blood	TaqMan® Open- Array™ Genotyping	FS ≤ 28%, decreased EF
Todorova et al. (66)	RC; 30	NR	53.1 (35–76)	Doxorubicin	BC	Peripheral blood	RT-PCR; Illumina HumanOmni BeadChip	Cardiac dysfunction, LVEF <55%
Garcia-Pavia et al. (67)	RC; 213	33/66; 0/73; 17/24	48.7 ± 17.1; 49.6 ± 10.8; 10.8 ± 5.6	Anthracyclines	Diverse cancers	Peripheral blood	Illumina TruSight Cardio Sequencing	Cardiomyopathy; LVEF <50%

*The characteristics of included studies. AML, acute myeloid leukemia; ALL, acute lymphoblastic leukemia; BC, breast cancer; EF, ejection fraction; CC, case-control; LVEF, left ventricular ejection fraction; LVFS, left ventricular fractional shortening; SF, shortening fraction; CHF, congestive heart failure; CTCAE, National Cancer Institute Common Toxicity Criteria; PC, prospective cohort; NCC, nested case control; NR, not reported; RC, retrospective cohort; RFLP, restriction fragment length polymorphism; ECG, echocardiography*.

The blood and buccal cells were the most commonly biological specimens used for the genotyping. Twenty-six studies used a single biological sample, including blood (24, 31–33, 35, 38, 40, 42–50, 57, 58, 62, 63, 65–67), bone marrow smear (36, 64) or buccal swab (34), while seven researches used more than one bio-specimens (23, 25, 34, 37, 51, 52, 54). Seven studies did not report the bio-specimens used for genotyping (39, 41, 53, 56, 59–61). Thirty studies used a single genotyping assay (24, 32, 33, 35, 37, 39–43, 45, 47–57, 59–65, 67) while the remaining studies used multiple genotyping assays (22, 24, 25, 31, 34, 36, 38, 44, 46, 50, 64). The most common detection technique were Sequenom MassARRAY (22, 25, 47, 57), (*n* = 4), Sequenom genotyping assay (42, 46, 48) (*n* = 3), TaqMan genotyping assay (22, 24, 36, 41, 44, 46, 50, 53, 55, 59, 65) (*n* = 11), pyrosequencing (24, 31, 46) (*n* = 3) and custom Illumina GoldenGate SNP genotyping assay (23, 39, 51) (*n* = 3).

The cardiotoxicity definition varied from study to study, with most studies using subjective results (*n* = 5), objective results (*n* = 11) or both (*n* = 24), while one did not define cardiotoxicity. However, most researches using subjective results defined cardiotoxicity as the signs and symptoms that required intervention. Furthermore, some studies have used shortened fraction (SF) or left ventricular ejection fraction (LVEF) as the objective indicators, but the critical points vary. For example, a cut-off value below 40–55% of the LVEF or reduce of more than 10–15% have been used. Five studies (24, 31, 35, 43, 64) also included the definition of electrocardiographic changes in cardiotoxicity, namely arrhythmias and electrocardiogram (ECG) abnormalities, while one study only detected the influences of anthracyclines on the QT intervals and arrhythmias.

### The Quality of the Reporting in the Studies

Among the studies reviewed (Table 2), there was only one study met the five main standard for reporting data from the genetic association studies in the STREGA guidelines. Most researches (*N* = 32) did not report the error rates or call rates related to the genotyping methods. Twenty-six researches did not indicate whether the genotyping was done in the batches or simultaneously. Twenty-nine studies did not provide any information on whether the population stratification was evaluated in our analysis.

**Table 2:** The quality assessment of reporting in each study (*N* = 41).

**Studies^***a***^**	**1**	**2**	**3**	**4**	**5**	**6**	**7**	**8**	**9**	**10**	**11**	**12**	**13**	**14**
*Genotyping methods and errors*														
Describe the laboratory methods: state the source and storage of DNA, the genotyping methods and the platforms	✓	✓	✓	✓	✓	✓	✓	✓	✓	✓	✓	✓	✓	✓
Describe the laboratory methods: state the error rates and call rates	✓	×	×	×	×	×	×	×	×	×	×	×	×	×
State the laboratory/center where the genotyping was done	✓	×	×	✓	×	×	✓	✓	✓	×	×	×	✓	×
Specify whether genotypes were assigned using all of the data from the study simultaneously or in smaller batches	✓	×	×	×	×	×	×	×	×	×	×	✓	✓	×
Report the numbers of individuals for whom genotyping was attempted and the numbers of individuals for whom genotyping was successful	✓	✓	✓	✓	✓	✓	✓	✓	✓	✓	✓	×	✓	✓
*Modeling population stratification*														
Describe any methods used to assess or address population stratification	✓	×	×	×	×	×	×	×	✓	×	×	×	×	×
*Modeling haplotype variation*														
Describe any methods used for inferring genotypes or haplotypes	✓	NA	NA	NA	NA	NA	NA	✓	NA	✓	NA	NA	NA	NA
*Hardy–Weinberg equilibrium*														
State whether the Hardy–Weinberg equilibrium was considered	✓	×	✓	✓	✓	✓	✓	✓	✓	✓	✓	✓	×	✓
*Replication*														
State if the study is the first report of a genetic association, a replication effort or both	✓	×	✓	✓	×	×	×	×	×	✓	✓	×	×	×
**Studies**^***a***^	**15**	**16**	**17**	**18**	**19**	**20**	**21**	**22**	**23**	**24**	**25**	**26**	**27**	**28**
*Genotyping methods and errors*														
Describe the laboratory methods: state the source and storage of DNA, the genotyping methods and the platforms	✓	✓	✓	✓	✓	✓	✓	✓	✓	✓	✓	✓	✓	✓
Describe the laboratory methods: state the error rates and call rates	×	×	✓	✓	×	×	×	×	×	✓	✓	×	✓	×
State the laboratory/center where the genotyping was done	×	✓	×	×	✓	×	×	✓	×	×	×	×	✓	×
Specify whether genotypes were assigned using all of the data from the study simultaneously or in smaller batches	×	✓	×	✓	✓	×	×	✓	×	✓	✓	×	×	✓
Report the numbers of individuals for whom genotyping was attempted and the numbers of individuals for whom genotyping was successful	✓	✓	✓	✓	✓	✓	✓	✓	✓	✓	✓	✓	✓	✓
*Modeling population stratification*														
Describe any methods used to assess or address population stratification	×	×	×	✓	×	×	✓	✓	×	×	✓	×	✓	×
*Modeling haplotype variation*														
Describe any methods used for inferring genotypes or haplotypes	NA	NA	NA	✓	NA	NA	✓	NA	NA	NA	✓	NA	✓	NA
*Hardy–Weinberg equilibrium*														
State whether the Hardy–Weinberg equilibrium was considered	✓	×	✓	✓	×	✓	✓	✓	×	×	✓	✓	✓	✓
*Replication*														
State if the study is the first report of a genetic association, a replication effort or both	×	×	×	×	✓	×	×	×	×	×	✓	×	×	✓
**Studies**^***a***^	**29**	**30**	**31**	**32**	**33**	**34**	**35**	**36**	**37**	**38**	**39**	**40**	**41**	
*Genotyping methods and errors*														
Describe the laboratory methods: state the source and storage of DNA, the genotyping methods and the platforms	✓	✓	✓	✓	✓	✓	✓	✓	✓	✓	✓	✓	✓	
Describe the laboratory methods: state the error rates and call rates	×	✓	×	×	×	×	×	×	×	×	✓	✓	×	
State the laboratory/center where the genotyping was done	✓	✓	✓	✓	✓	×	✓	×	✓	✓	×	×	×	
Specify whether genotypes were assigned using all of the data from the study simultaneously or in smaller batches	×	✓	✓	×	✓	×	✓	✓	✓	×	×	×	×	
Report the numbers of individuals for whom genotyping was attempted and the numbers of individuals for whom genotyping was successful	✓	✓	✓	✓	✓	✓	✓	✓	✓	✓	✓	✓	✓	
*Modeling population stratification*														
Describe any methods used to assess or address population stratification	×	×	×	×	✓	✓	✓	×	✓	×	×	×	✓	
*Modeling haplotype variation*														
Describe any methods used for inferring genotypes or haplotypes	NA	NA	NA	NA	NA	✓	NA	NA	NA	NA	✓	NA	NA	
*Hardy–Weinberg equilibrium*														
State whether the Hardy–Weinberg equilibrium was considered	×	✓	✓	×	✓	×	✓	×	✓	×	✓	×	×	
*Replication*														
State if the study is the first report of a genetic association, a replication effort or both	×	×	×	✓	✓	×	✓	×	×	×	×	×	✓	

a *1. Wojnowski et al., 2005; 2. Weiss et al., 2006; 3. Beauclair et al., 2007; 4. Blanco et al., 2008; 5. Rossi et al., 2009; 6. Rajić et al., 2009; 7. Blanco et al., 2012; 8. Semsei et al., 2012; 9. Visscher et al., 2012; 10. Cascales et al., 2012; 11. Volkan-Salanci et al., 2012; 12. Lubieniecka et al., 2012; 13. Kitagawa et al., 2012; 14. Windsor et al., 2012; 15. Roca et al., 2013; 16. Lipshultz et al., 2013; 17. Armenian et al., 2013; 18. Lubieniecka et al., 2013; 19. Sachidanandam et al., 2013; 20. Vivenza et al., 2013; 21. Visscher et al., 2013; 22. Wang et al., 2014; 23. Wasielewski et al., 2014; 24. Krajinovic et al., 2015; 25. Visscher et al., 2015; 26. Peña et al., 2015; 27. Aminkeng et al., 2015; 28. Reichwagen et al., 2015; 29. Vulsteke et al., 2015; 30. Stanton et al., 2015; 31. Hertz et al., 2016; 32. Reinbolt et al., 2016; 33. Wang et al., 2016; 34. Schneider et al., 2017; 35. Ruiz-Pinto et al., 2017; 36. Pop-Moldovan et al., 2017; 37. Ruiz-Pinto et al., 2017; 38. Huang et al., 2017; 39. Sági et al., 2018; 40. Todorova et al., 2018; 41. Garcia-Pavia et al., 2019*.

### Chemotherapy-Induced Cardiotoxicity (CIC) and Susceptibility Genes

**Figure 2:**
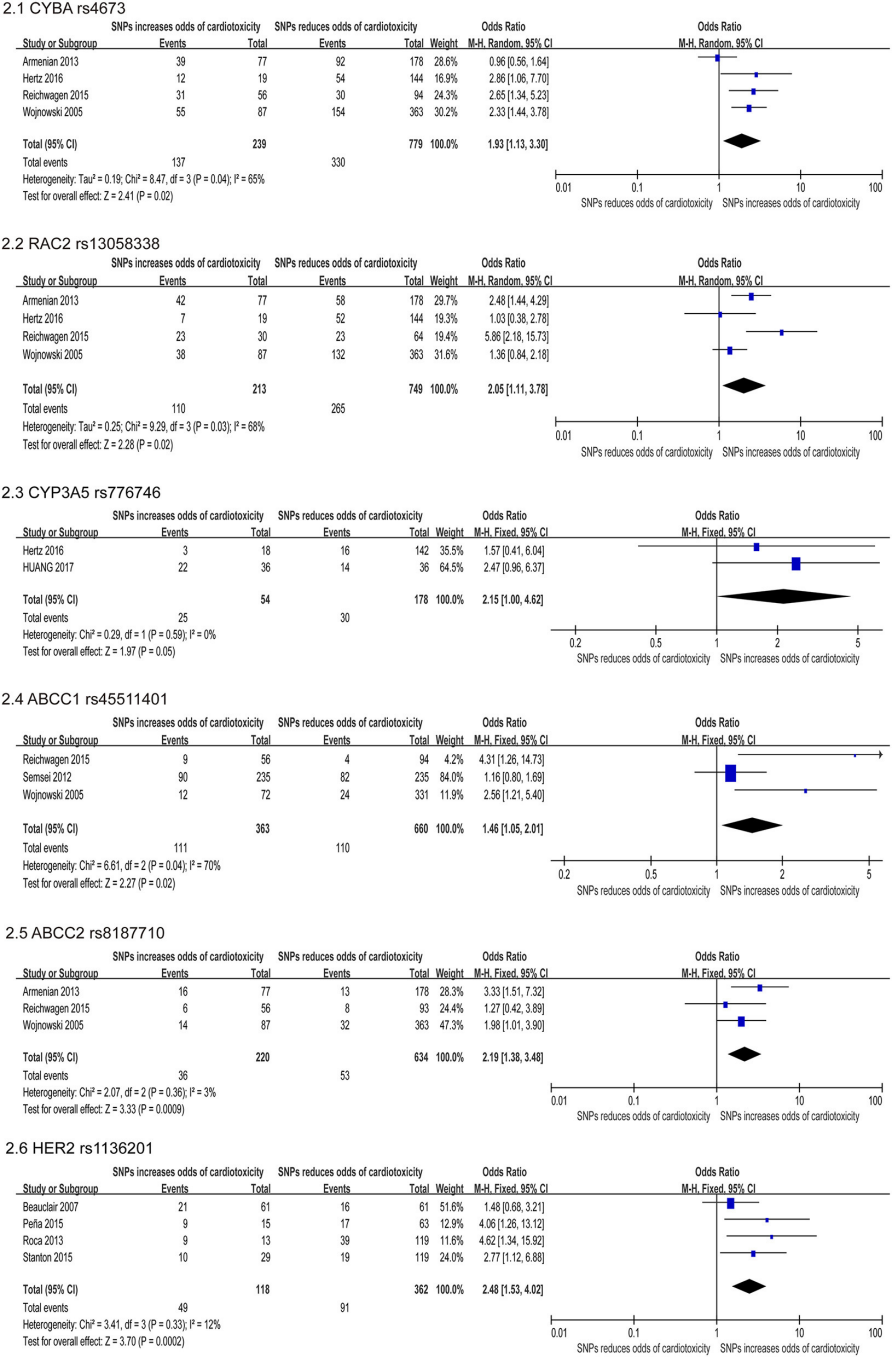
Forest plot of meta-analysis for 6 SNPs. Six variants, CYBA rs4673, RAC2 rs13058338, CYP3A5 rs776746, ABCC1 rs45511401, ABCC2 rs8187710, and HER2 rs1136201 are significantly increased the odds for chemotherapy induced cardiotoxicity.

**Figure 3:**
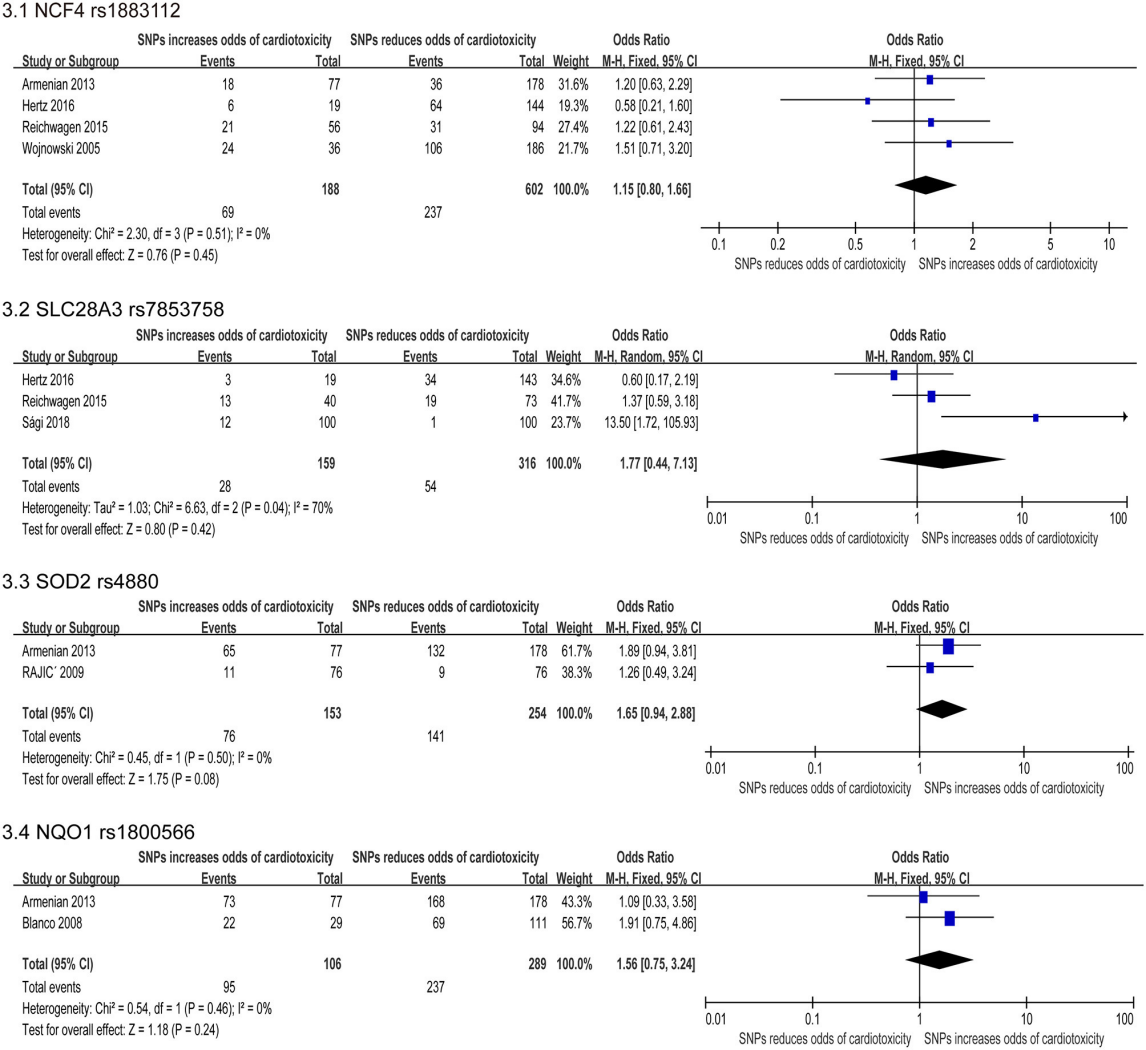
Forest plot of meta-analysis for 4 SNPs. Four variants, NCF4 rs1883112, SLC28A3 rs7853758, SOD2 rs4880, and NQO1 rs1800566, are not statistically significant for chemotherapy induced cardiotoxicity.

**Figure 4:**
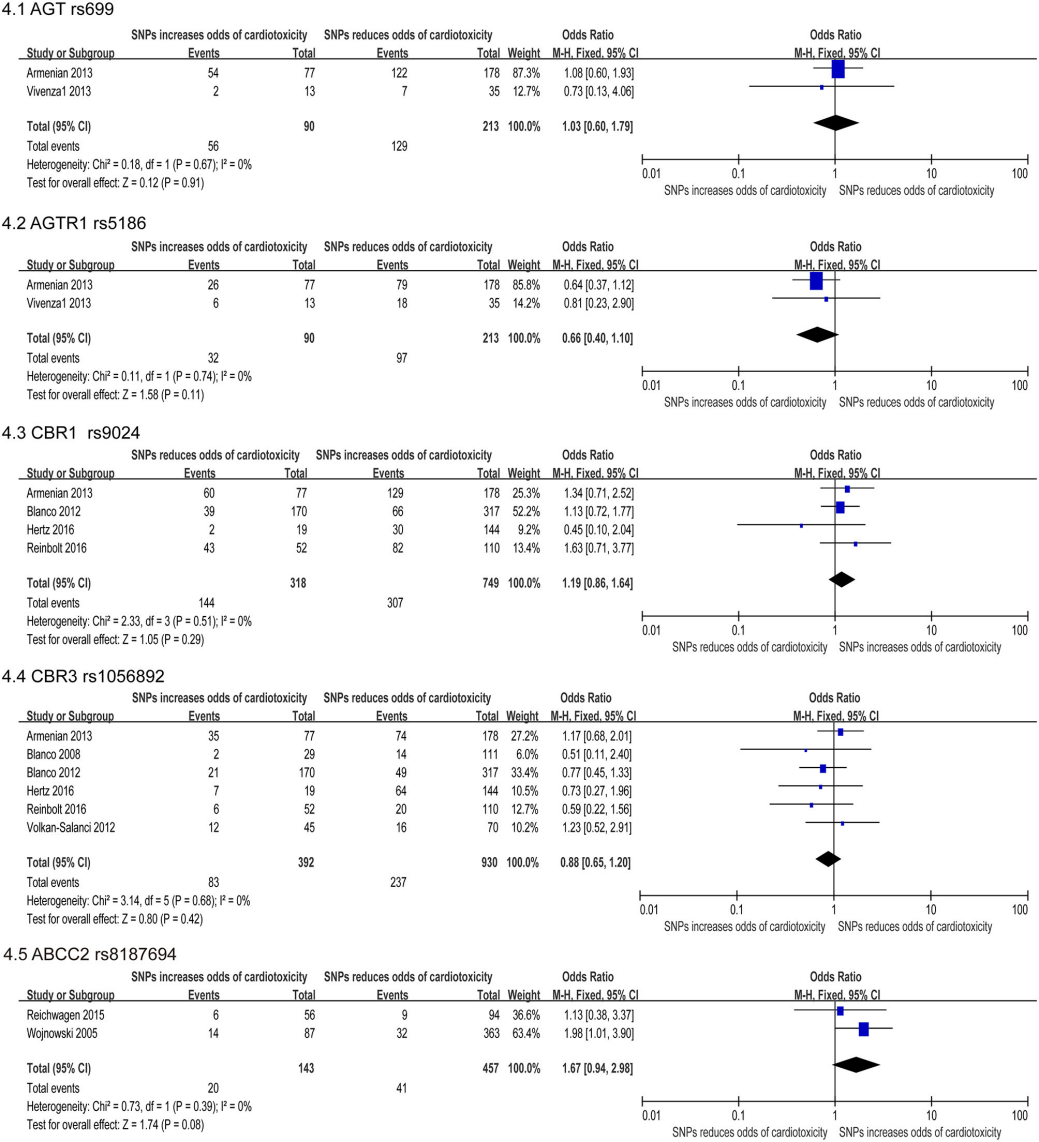
Forest plot of meta-analysis for five SNPs. Five variants, AGT rs699, AGTR1 rs5186, CBR1 rs9024, CBR3 rs1056892, and ABCC2 rs8187694, are not statistically significant for chemotherapy induced cardiotoxicity.

Our study reported a total of 154 SNPs involving eighty-eight genes (Supplementary Table 1). Most of the research has focused on genetic variations linked to chemical synthesis or heart function. Accessable data was found in 17 studies out of the included 41 studies and 14 SNPs were consistently detected. Then these 14 SNPs were subjected to our quantitative analysis (Figures 2–4). And we searched for these six variants in the protein-coding region of the gene in the Pubmed (Table 3). The genes included in the meta-analysis are discussed below.

**Table 3:** The 6 variants, annotations and meta-analysis OR and *p-*value.

**Gene**	**SNP**	**Variant type**	**Alleles**	**Chromosome**	**Functional consequence**	**Clinical significance**	**Odds ratio**	***p-*value**
CYBA	rs4673	SNV	A>G,T	16:88646828 (GRCh38)	Coding sequence variant	Benign, likely-pathogenic	1.93	0.02
RAC2	rs13058338	SNV	T>A,G	22:37236730 (GRCh38)	Intron variant	Not report	2.05	0.02
CYP3A5	rs776746	SNV	T>C	7:99672916 (GRCh38)	Intron variant	Benign, drug-response	2.15	0.05
ABCC1	rs45511401	SNV	G>T	16:16079375 (GRCh38)	Coding sequence variant	Not report	1.46	0.02
ABCC2	rs8187710	SNV	G>A	10:99851537 (GRCh38)	Coding sequence variant	Likely-benign	2.19	0.0009
Her2	rs1136201	SNV	A>G,T	17:39723335 (GRCh38)	Coding sequence variant	Benign, not-provided	2.48	0.0002

#### Cytochrome b-245, Alpha Polypeptide (CYBA) Gene

The CYBA gene encodes a major component of the phagocytes microbicidal oxidase system. Meanwhile, our study confirmed six studies that assessed the correlation between CYBA rs4673 and cardiotoxicity, and four studies (22, 24, 31, 47) were included in quantitative analysis. In our study, this SNP was found to increase the risk of developing cardiotoxicity (OR: 1.93; 95% CI: 1.13–3.30; *p* = 0.04) with high heterogeneity (I^2^ = 65%) (Figure 2.1). We used the random effects model and excluded individual studies to conduct sensitivity analysis to account for the heterogeneity, and these results show that excluding the study of Armenian et al. can alter heterogeneity (I^2^ = 0%).

#### Ras-Related C3 Botulinum Toxin Substrate 2 (RAC2) Gene

The RAC2 gene encodes proteins that regulate a variety of processes including secretion, phagocytosis, cell polarization, and ROS production. Four of the six studies indicated that the SNP rs13058338 on the RAC2 obviously increased the risk of cardiotoxicity (22, 24, 31, 47). We used the random effects model and the analysis of this variant in four studies suggested that the RAC mutation significantly increased the risk of cardiotoxicity by nearly twice (OR: 2.05; 95% CI: 1.11–3.78; *p* = 0.02), but with moderate heterogeneity (I^2^ = 68%) (Figure 2.2). Sensitivity analyses were conducted to explore the potential sources of heterogeneity, but these results did not change substantially [I^2^ = 74% (47), 74% (22), 60% (24), 66% (31)].

#### Cytochrome P450 Family 3 Subfamily A Member 5 (CYP3A5) Gene

The CYP3A5 gene is involved in the metabolism and clearance of daunorubicin (DNR), but the activity of CYP3A5 is impacted on the gene polymorphism, which has individual differences (68). Meta-analysis of two studies (22, 64) reported that the rs776746 on CYP3A5 increased cardiotoxicity risk, including a total of 232 patients (54 in the case group and 178 in the control group). Using the FE model, our study suggested that the missense mutation was related to an obviously increase in the risk of cardiotoxicity [OR = 2.15 [95% CI (1.00–4.62)], *P* = 0.05] (Figure 2.3).

#### ATP Binding Cassette (ABC) Genes

The ABC transporter gene encodes a superfamily of transmembrane proteins that can use adenosine triphosphate to actively transport substrates including doxorubicin through the membrane (69). Meta-analysis of three studies (24, 31, 47) reported that the ABCC1 rs45511401 increased cardiotoxicity risk, including a total of 1,023 patients (363 in the case group and 660 in the control group). Using the random effects model, we found that missense mutations were related to a significant increase in risk [OR = 1.46 [95% CI (1.05–2.01)], *P* = 0.02] with moderate heterogeneity (I^2^ = 70%) (Figure 2.4). The study conducted by Semsei et al. (38) were excluded, changing the results and addressing heterogeneity (I^2^ = 0%). And three studies (24, 31, 38) indicated that the ABCC2 rs8187710 significantly augmented the risk of cardiotoxicity (OR: 2.19; 95% CI: 1.38–3.48; *p* = 0.0009) (Figure 2.5).

#### Human Epidermal Growth Factor Receptor 2 (HER2) Gene

The HER2 is a proto-oncogene that encodes transmembrane proteins that have tyrosine kinase activity, but have not been identified as the physiological ligand. Four included studies (33, 45, 55, 58) revealed Ile655Val rs1136201 on HER2 significantly increase risk for cardiotoxicity, including a total of 480 patients (118 in the case group and 362 in the control group). Using the FE model, the result indicated that the missense mutation was related to a significant increase in the risk of cardiotoxicity [OR = 2.48 [95% CI (1.53–4.02)], *P* = 0.0002] with low heterogeneity (I^2^ = 12%) (Figure 2.6).

#### Neutrophil Cytosolic Factor 4 (NCF4) Gene

The NCF4 gene encode the p40phox subunit of the NAD(P)H oxidase (NOX) (70). The rs1883112 polymorphism of NCF4 promoter blocks the oxidase activation of the enzyme, thus reducing the formation of active oxidant intermediates (71). Three (22, 24, 31, 47) of the six researches (22, 24, 31, 35, 39, 47) studied the roles of SNP rs1883112, and found that this SNP was related to cardiotoxicity, but the combined effect of this synonymous substitution indicated no significant relevancy (OR: 1.13; 95% CI: 0.72–1.77; *p* = 0.59) (Figure 3.1).

#### Solute Carrier Family 28 Member 3 (SLC28A3) Gene

It has been previously reported that SLC28A3 (rs7853758) has cardioprotective effects in the multiple patient cohorts with an odds ratio of 0.35–0.42 (39, 51). We identified five studies that assessed the association between rs7853758 missense SNP on SLC28A3 and cardiotoxicity, and three studies (22, 24, 65) were included in the quantitative analysis. We used the random effects model. But the SNP rs7853758 was not statistically significant difference (OR: 1.77; 95% CI: 0.44–7.13; *p* = 0.42) (Figure 3.2) with high heterogeneity (I^2^ = 70%). Therefore, we excluded individual studies to conduct sensitivity analyses to account for the heterogeneity, and the result suggested no significant differences between the selected studies.

#### Superoxide Dismutase II (SOD2) Gene

SOD2 exists in the mitochondria and metabolizes superoxide radicals formed when anthracycline compounds are oxidized to hydrogen peroxide. Meta-analysis of two included studies (36, 47) revealed rs4880 on SOD2 significantly increase risk for cardiotoxicity including a total of 407 participants (153 in the case group and 254 in the control group). But the FE model indicated that the missense mutation was not statistically significant [OR = 1.65 [95% CI (0.94–2.88)], *P* = 0.08] (Figure 3.3).

#### NAD(P)H Quinone Dehydrogenase1 (NQO1) Gene

The NQO1 gene is involved in the protection of intracellular OS, and many pro-oxidant drugs induced basic NQO1 activity (34). Two included studies (34, 47) revealed SNP rs1800566 on NQO1 significantly increase risk for cardiotoxicity, including a total of 395 patients (106 in the case group and 289 in the control group). Using the FE model, the result indicated that the missense mutation was not statistically significant [OR = 1.56 [95% CI (0.75–3.24)], *P* = 0.24] (Figure 3.4).

#### Angiotensinogen (AGT) Gene

Association between AGT P.m.ET235THr gene polymorphism and cardiovascular disease concluded that there was a positive correlation between essential hypertension (72, 73) and myocardial infarction (74). Two included studies (44, 47) revealed SNP rs699 AGT significantly increase risk for cardiotoxicity, including a total of 303 participants (90 in the case group and 213 in the control group). The FE model indicated that the missense mutation was not statistically significant [OR = 1.93 [95% CI (0.60–1.79)], *P* = 0.91] (Figure 4.1).

#### Angiotensin II Type-1 Receptor (AGTR1) Gene

Angiotensin II is the main ligand of AGTR1A, which adjusts the intravascular volume and the blood pressure (75). And functional polymorphisms in the ACE, AGT, and AGTR1 can impact the expression or the function of encoded proteins, and are related to stroke, coronary heart disease, vascular dysfunction and diabetes (76). Meta-analysis of two included studies (47, 50) revealed SNP rs5186 significantly increase risk for cardiotoxicity, including a total of 303 patients (90 in the case group and 213 in the control group). But the FE model indicated that the missense mutation was not statistically significant difference [OR = 0.66 [95% CI (0.40–1.10)], *P* = 0.11] (Figure 4.2).

#### Carbonyl Reductases (CBR) Genes

The enzyme encoded by the CBR genes catalyzes the reduction of the endogenous aliphatic aldehydes and ketones as well as various xenobiotic, and thus has a cardioprotective effect against cardiotoxicity. Four SNPs on CBRs were studied, one on carbonyl reductase 1 (CBR1) gene and three on carbonyl reductase 3 (CBR3) gene. However, two SNPs, rs9024 of CBR1 (22, 37, 47, 59) and rs1056892 of CBR3 (22, 34, 37, 41, 47, 59) were not statistically significant (OR: 1.19; 95% CI: 0.86–1.64 and 0.88; 0.65–1.20, respectively) (Figures 4.3,4.4).

#### ATP Binding Cassette (ABC) Gene

Two studies (24, 31) reported that the ABCC2 rs8187694 increased cardiotoxicity risk, including a total of 600 patients (457 in the case group and 143 in the control group). Using the FE model, we found no statistically significant difference (OR: 1.67; 95% CI: 0.94–2.98; *p* = 0.08) with low heterogeneity (I^2^ = 0%) (Figure 4.5).

### Sensitivity Analyses

**Figure 5:**
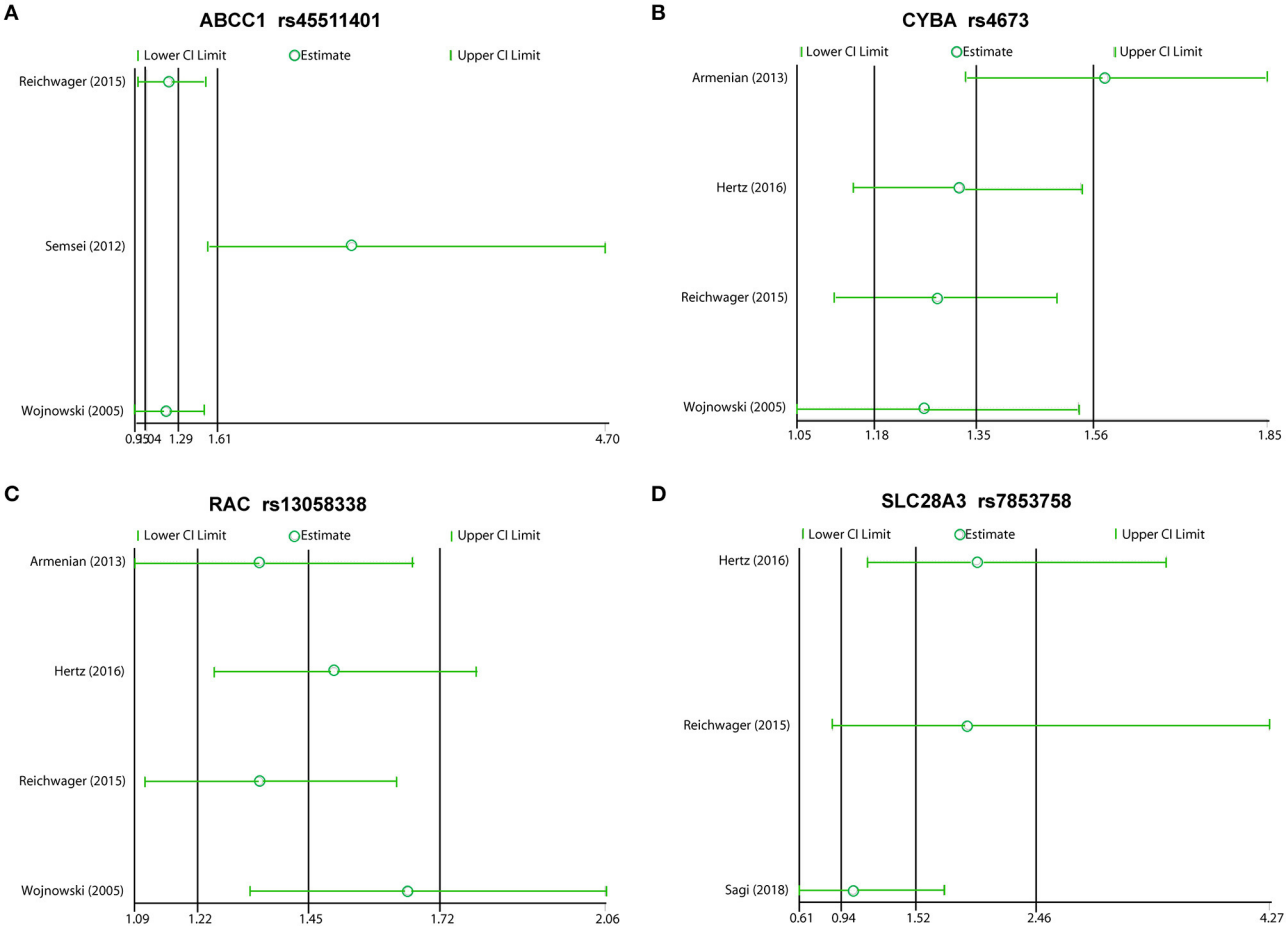
Sensitivity analyses plot of ABCC1 **(A)**, CYBA **(B)**, RAC **(C)**, and SLC28A3 **(D)**.

We excluded each study in order, and the rest reported inconsistent results between ABCC1 and CYBA. In this meta-analysis, the studies that included Semsei et al. (38) and Armenia et al. (47) obviously distorted these results, suggesting that the two reports may be statistically unstable (Figures 5A,B). Other results showed consistent results (Figures 5C,D).

## Discussion

Our study analyzed the association between genetic polymorphism and CIC. We included a total of 41 studies that screened 88 different genes. Gene variation may promote OS, metabolic disorders, mitochondrial dysfunction, calcium overload, myocardial fibrosis, sarcoplasmic reticulum structure and function destruction, cardiomyocyte autophagy and apoptosis in CIC (77, 78). The results of our meta-analysis showed that the polymorphisms in six (6.8%) of eighty-eight genes were obviously associated with the risk of cardiotoxicity in patients receiving chemotherapy.

For genes with positive associations, mechanism studies have indicated that these alleles can alter the encoded proteins expression or activity, leading to cardiotoxicity. The ABCC1 transporter plays an important function in the OS, and is involved in maintaining the adequate levels of glutathione, which is essential for ROS defense. In addition, ABCC1 also requires glutathione to transport anthracycline antibiotics (79–81), which may also affect the OS response induced by anthracycline antibiotics (82). The ABCC2 gene encoded proteins involved in the efflux of substances from cells, and ABCC2 mutation obviously decrease the ATPase-activity, leading to reduce in the efflux activity resulting in the accumulation of anthracyclines in cells (83). CYBA encodes p22phox, which is one of the two subunits of the NOX located in the cell membrane. And the reduced activity of inherited NOX may lead to impaired ROS defense capacity, thus increasing ROS levels under chemotherapy exposure. Similarly, RAC2 encoded by the RAC2 gene is a mitochondrial protein required for the electron transfer reaction of NOX (84) during OS formation (85). Genetic alteration leads to mitochondrial dysfunction, which leads to an increase in ROS production and ultimately cardiomyocyte damage. There is also evidence that the CYP3A5-mediated oxidative metabolism of anthracyclines may induce drug-induced cardiotoxicity by generating OS (86). Taken together, these gene mutations are believed to cause cardiotoxicity on account of the accumulation of chemotherapy and excessive ROS in cardiomyocytes.

Some other genes were also observed to be associated with CIC, such as HER2-Ile655Val rs1136201. The most studied germline polymorphism in the clinical level is associated with the transmembrane domain of HER2 protein 655 A>G Ile/Val (87, 88), which may be related to a high risk of BC (89). The Val allele presence may make cardiomyocytes especially dependent on HER2-Ile655Val rs1136201 signaling and the highly sensitive to trastuzumab (33). This mechanism of cardiotoxicity induced by trastuzumab is unknown, however, HER2/neu has been shown to be critical for cardiomyocytes in animal model. Our meta-analysis also confirmed the role of HER2-Ile655Val polymorphism as a genetic predictor of cardiac toxicity induced by trastuzumab in the BC patients.

There are some limitations to this study that deserve discussion. Firstly, the sample size of the study was small. This is particularly important for genome-wide association study (GWAS), with some literature advocating 10,000 cases to gain sufficient statistical power to detect causality through meta-analysis and data aggregation. Sixty one percentage of the included studies had fewer than 200 people. Secondly, there is selection bias in this study. Most literature used a retrospective method, usually by convenience sampling to recruit patients who were still in the hospital system, thereby further limiting the choice to participants who are still alive. In addition, the analysis was based on previous reports, which may not be complete or accurate. Finally, due to the lack of raw data, studies on drug dosage protocols and study periods varied widely. In addition, patient cohorts were often heterogeneous in terms of disease, drug dose, drug route, and administration, all of which may confuse toxicity associated with the target drug.

## Conclusions

This study suggests that the polymorphisms in multiple pharmacogenetic in the biochemical and cardiotoxicity pathways may be predictors of CIC. However, for limited quantitative analysis, the evidences are limited and too diverse. Further researches are needed to produce reliable genetic predictors of CIC in order to achieve the goal of individualized chemotherapy.

## Data Availability Statement

The original contributions presented in the study are included in the article/Supplementary Material, further inquiries can be directed to the corresponding author/s.

## Author Contributions

YX, HS, and SD designed the idea for drafting this review. XY, GL, MG, and AB collected the documents and wrote the paper. TY, QD, CZ, WL, and NA contributed to discussion. FY, HP, PW, CL, YGo, and YGa revised and edited the review. All authors commented on the manuscript.

## Conflict of Interest

The authors declare that the research was conducted in the absence of any commercial or financial relationships that could be construed as a potential conflict of interest.
