# Supplementary material for: Potential Gene Association Studies of Chemotherapy-Induced Cardiotoxicity: A Systematic Review and Meta-Analysis
Source: Front Cardiovasc Med. 2021 Jun 4;8:651269. doi: 10.3389/fcvm.2021.651269 (PMC8213036; doi:10.3389/fcvm.2021.651269)
Supplement: Supplementary file 1 [file Table_1.docx]

**Supplementary Table 1: Genetic polymorphisms associated with cardiotoxicity.**

| Gene | Nucleotide change | SNP rsID | Amino acid change | Association^2^ | Study  (Name, Year) |
| --- | --- | --- | --- | --- | --- |
| ABCA1 | 106738433G>A | rs3887137 | None | Significant correlation | Visscher et al.,2015 |
| ABCB1 | g.87138532A>C | rs2235047 | None | No significant relationship | Visscher et al., 2013 |
|  | g.87138645A>G | rs1045642 | Ile1145 |  | Rossi et al., 2009 |
|  |  |  |  |  | Hertz et al., 2016 |
|  | g.87179809C>T | rs2229109 | Ser400Asn |  | Rossi et al., 2009 |
|  | g.87179601A>G | rs1128503 | Gly412 |  | Hertz et al., 2016 |
|  | g.87160618G>T | rs2032582 | Ser893Thr |  |  |
| ABCB4 | g.87073775T>G | rs1149222 | None | Risk variant | Visscher et al., 2012 |
|  |  |  |  | No significant relationship | Visscher et al., 2013 |
|  |  |  |  |  | Hertz et al., 2016 |
|  |  |  |  |  | Sachidanandam et al., 2012 |
|  | g.87105795A>G | rs4148808 | None | Significant correlation | Visscher et al., 2013 |
|  |  |  |  | No significant relationship | Hertz et al., 2016 |
| ABCB11 | 169479422A>G | rs10497346 | None | Significant correlation | Visscher et al., 2015 |
| ABCC1 | g.15983430G>A | rs215060 | None | No significant reduction in LVFS after chemotherapy | Semsei et al., 2012 |
|  | g.15991778T>C | rs246219 | None |  |  |
|  | g.16108028G>A | rs11864374 | None |  |  |
|  | g.16120105G>A | rs6416666 | None |  |  |
|  | g.16141824C>T | rs3743527 | None | TT genotype is associated with lower mean LVFS |  |
|  | g.16044465T>C | rs246221 | Val275 | TT/TC genotypes are associated with lower mean LVFS | Semsei et al., 2012 |
|  |  |  |  |  |  |
| **Supplementary Table 1: Genetic polymorphisms associated with cardiotoxicity.** (continued) | | | | | |
| Gene | Nucleotide change | SNP rsID | Amino acid change | Association^2^ | Study  (Name, Year) |
|  |  |  |  | SNP is associated with an asymptomatic LVEF | Vulsteke et al., 2015 |
|  | g16079375G>T | rs45511401 | Gly671Val | No significant reduction in LVFS after chemotherapy | Semsei et al., 2012 |
|  |  |  |  | No significant relationship | Reichwagen et al., 2015 |
|  |  |  |  | GT/TT genotypes are associated with cardiotoxicity | Wojnowski et al., 2005 |
|  | g.6093318C>T | rs4148358 | None | No significant reduction in LVFS after chemotherapy | Semsei et al., 2012 |
|  | g.16076620G>T | rs4148350 | None | Risk variant | Visscher et al., 2012 |
|  |  |  |  | No significant relationship | Hertz et al., 2016 |
| ABCC2 | g.101611294G>A | rs8187710 | Cys1515Tyr | At risk genotype GA/AA | Armenian et al., 2013 |
|  |  |  |  | No significant relationship | Wojnowski et al., 2005 |
|  |  |  |  |  | Reichwagen et al., 2015 |
|  | T>A | rs8187694 | Val1188Glu |  | Visscher et al., 2012 |
|  |  |  |  |  | Reichwagen et al., 2015 |
|  |  |  |  | TA/AA genotypes are associated with cardiotoxicity | Wojnowski et al., 2005 |
|  |  | rs3740066 |  | Significant correlation | Sági et al., 2005 |
|  | g.101595996T>A | rs17222723 | Val1085Glu | No significant relationship | Rossi et al., 2009 |
| ABCC5 | g.183737356A>T | rs7627754 | None | Homozygote variant vs heterozygote + homozygote wt, EF reduction of 12% and SF reduction of 8% | Krajinovic et al., 2015 |
| **Supplementary Table 1: Genetic polymorphisms associated with cardiotoxicity.** (continued) | | | | | |
| Gene | Nucleotide change | SNP rsID | Amino acid change | Association^2^ | Study  (Name, Year) |
| ABCC6 | g.16150272T>C | rs212097 | None | No significant reduction in LVFS after chemotherapy | Semsei et al., 2012 |
| ABCC9 | g.22017395G>C | rs201223488 | Pro739Ala | No significant relationship | Wasielweski et al., 2014 |
|  | 21908424G>C | rs11046217 | None | Significant correlation | Visscher et al., 2015 |
| ABCC10 | 43468329A>G | rs1214763 | None | Significant correlation | Visscher et al., 2015 |
| ABCG2 | g.89052323G>T | rs2231142 | Gln141Lyn | No significant relationship | Rossi et al., 2009 |
|  | g.89061114C>T | rs2231137 | Val12Met |  |  |
| ACE | Ins/Del | rs4340 |  | Del/Del + Del/Ins vs Ins/Ins | Vivenza et al., 2013 |
|  | g.61566031G>A | rs4343 | Thr202 | At risk genotype AG/GG | Armenian et al., 2013 |
| ADH7 | g.100333267G>A | rs729147 | None | Significant correlation | Visscher et al., 2013 |
| ADRB2 | g.148206440G>A | rs1042713 | Gly16Arg | At risk genotype GG | Armenian et al., 2013 |
| AGT | g.230845977G>A | rs4672 | Thr174Met | Homozygote variant vs heterozygote + homozygote wt | Vivenza et al., 2013 |
|  | g.230845794A>G | rs699 | Met235Thr |  |  |
|  |  |  |  | At risk genotype GA/AA | Armenian et al., 2013 |
| AGTR1 | g.148459988A>C | rs5186 | None | Homozygote variant + heterozygote vs homozygote wt | Vivenza et al., 2013 |
|  |  |  |  | At risk genotype CA/AA | Armenian et al., 2013 |
| AKR1A1 | g.46032311A>G | rs2229540 | Asn52Ser | No significant association | Lubieniecka et al., 2012 |
| AKR1C4 | g.5260682C>G | rs17134592 | Leu311Val | No significant association | Lubieniecka et al., 2012 |
|  | g.5244295G>A | rs7083869 | None | SNP is associated with cardiotoxicity | Lubieniecka et al., 2013 |
|  | g.5244441A>G | rs2151896 | None |  |  |
| AKR7A2 | g.19635011C>T | rs1043657 | Ala142Thr | No significant association | Lubieniecka et al., 2012 |
|  |  |  |  |  |  |
| **Supplementary Table 1: Genetic polymorphisms associated with cardiotoxicity.** (continued) | | | | | |
| Gene | Nucleotide change | SNP rsID | Amino acid change | Association^2^ | Study  (Name, Year) |
| CAT | g.34438684C>T | rs1001179 | None | No significant correlation between SNP and cardiac damage | Rajic et al., 2009 |
|  | g.34439157C>T | rs10836235 | None | Heterozygote is correlated with late cardiac damage |  |
| CBR1 | g.37445313G>A | rs9024 | None | Significant correlation | Blanco et al., 2012 |
|  |  |  |  | No significant relationship | Hertz et al., 2016 |
|  |  |  |  |  | Reinbolt et al., 2016 |
|  |  |  |  |  | Sachidonandam et al., 2012 |
|  |  |  |  | At risk genotype GG | Armenian et al., 2013 |
| CBR3 | g.37518706G>A | rs1056892 | Val244Met | Significant correlation | Blanco et al., 2008 |
|  |  |  |  | No significant association | Lubieniecka et al., 2012 |
|  |  |  |  | At risk genotype GG | Armenian et al., 2013 |
|  |  |  |  | No significant relationship | Visscher et al., 2015 |
|  |  |  |  | Significant correlation | Blanco et al., 2012 |
|  |  |  |  | Presence of A allele is associated with deterioration in cardiac function. | Volkan-Salanci et al., 2012 |
|  |  |  |  | Significant correlation | Hertz et al., 2016 |
|  |  |  |  | No significant relationship | Reinbolt et al., 2016 |
|  | g.37507501G>A | rs8133052 | Cys4Tyr |  | Lubieniecka et al., 2012 |
|  | g.37512565A>G | rs10483032 | None | SNP is associated with LVEF drop | Lubieniecka et al., 2013 |
| CELF4 | g.37497065G>A | rs1786814 | None | GG genotype is associated with anthracycline-related ardiomyopathy | Wang et al., 2016 |
| COL1A2 | g.93881175C>G | rs42524 | Pro549Ala | Significant correlation | Visscher et al., 2015 |
| **Supplementary Table 1: Genetic polymorphisms associated with cardiotoxicity.** (continued) | | | | | |
| Gene | Nucleotide change | SNP rsID | Amino acid change | Association^2^ | Study  (Name, Year) |
| CYBA | 242C>T | rs4673 | His72Tyr | No significant association | Visscher et al., 2012 |
|  |  |  |  |  | Reichwagen et al., 2015 |
|  |  |  |  |  | Hertz et al., 2016 |
|  |  |  |  | CT/TT genotypes are associated with cardiotoxicity | Wojnowski et al., 2005 |
|  |  |  |  | Significant correlation | Rossi et al., 2009 |
|  |  |  |  | At risk genotype GA/AA | Armenian et al., 2013 |
| CYP1A2 | g.74746892T>G | rs2069522 | None | SNP is associated with LVEF drop | Lubieniecka et al., 2013 |
|  | g.74749000T>G | rs2069526 | None |  |  |
|  | g.74753351T>C | rs4646427 | None |  |  |
| CYP2B6 | g.41023115G>A | rs7255904 | None | SNP is associated with LVEF drop | Lubieniecka et al., 2013 |
| CYP2F1 | g.41113670T>C | rs1709115 | None | SNP is associated with LVEF drop | Lubieniecka et al., 2013 |
| CYP2J2 | 60087084A>C | rs2294950 | None | Significant correlation | Visscher et al., 2015 |
| CYP3A4 | g.99366316G>A | rs35599367 | None | No significant relationship | Hertz et al., 2016 |
| CYP3A5 | g.99270539C>T | rs776746 | None | No significant relationship | Hertz et al., 2016 |
|  |  |  |  | Significant correlation | Sági et al., 2016 |
|  |  |  |  |  | HUANG et al., 2017 |
| CYP4B1 | g.47265776A>G | rs837400 | None | SNP is associated with LVEF drop | Lubieniecka et al., 2013 |
|  | g.47283505G>T | rs4646495 | None |  |  |
| CYP4F11 | g.15906807G>A | rs8112732 | None | SNP is associated with LVEF drop | Lubieniecka et al., 2013 |
|  | g.15912567A>G | rs12610962 | None |  |  |
|  | g.15913964A>G | rs2072270 | None |  |  |
|  | g.15921833A>C | rs11086012 | None | No significant association |  |
| **Supplementary Table 1: Genetic polymorphisms associated with cardiotoxicity.** (continued) | | | | | |
| Gene | Nucleotide change | SNP rsID | Amino acid change | Association^2^ | Study  (Name, Year) |
|  | g.15906196G>A | rs2108623 | None | No significant relationship | Visscher et al., 2013 |
| CYP11B2 | g.143999600A>G | rs1799998 | None | Homozygote variant + heterozygote vs homozygote wt | Vivenza et al., 2013 |
| E1199 | NR | rs28714259 | NR | Significant correlation | Schneider et al., 2017 |
| FMO2 | 37236730T>A | rs2020870 | Asp36Gly | Protective variant | Visscher et al., 2012 |
| FMO3 | g.171080080G>A | rs1736557 | Val257Met | Significant correlation | Visscher et al., 2013 |
| GPX3 | 150395291G>C | rs2233302 | None | Significant correlation | Visscher et al., 2015 |
| GPR35 | c.758C >T | rs12468485 | p.Thr253Met | Significant correlation | Ruiz-Pinto et al., 2017 |
| GSTA1 | NR | NR | NR | No significant relationship | Weiss et al., 2006 |
| GSTA2 | 52725690C>G | rs2180314 | Ser112Thr | Significant correlation | Visscher et al., 2015 |
| GSTM1 | NR | NR | NR | No significant relationship | Weiss et al., 2006 |
|  | NR | NR | NR |  | Rajic et al., 2009 |
|  | NR | NR | NR |  | Rossi et al., 2009 |
|  | NR | NR | NR | Present (+/- and +/+) vs Null (-/-) | Vivenza et al., 2013 |
| GSTT1 | NR | NR | NR | No significant relationship | Weiss et al., 2006 |
|  | NR | NR | NR |  | Rajic et al., 2009 |
|  | NR | NR | NR | Present (+/- and +/+) vs Null (-/-) | Vivenza et al., 2013 |
| GSTM3 | 110075064 A>G | rs12059276 | None | Significant correlation | Visscher et al., 2015 |
| GSTP1 | g.67585218A>G | rs1695 | Ile105Val | Presence of G allele is associated with cardiotoxicity | Volkan-Salanci et al., 2012 |
|  |  |  |  | Significant correlation | Rossi et al., 2009 |
|  |  |  |  | Variants are associated with increased risk of cardiotoxicity | Windsor et al., 2012 |
| **Supplementary Table 1: Genetic polymorphisms associated with cardiotoxicity.** (continued) | | | | | |
| Gene | Nucleotide change | SNP rsID | Amino acid change | Association^2^ | Study  (Name, Year) |
|  |  |  |  | Homozygote variant + heterozygote vs homozygote wt | Vivenza et al., 2013 |
|  | g.67586108C>T | rs1138272 | Ala114Val | No significant relationship | Rossi et al., 2009 |
| HAS3 | g.69109674A>C | rs2232228 | Ala93 | Presence of allele A increase the risk of cardiomyopathy | Wang et al., 2014 |
| HER2 | NR | NR | Ile655Val | Significant correlation | Beauclair et al., 2007 |
|  |  |  |  |  | Roca et al., 2013 |
|  | 655 A>G | rs1136201 | Ile655Val |  | Peña et al., 2015 |
|  | NR | rs1136201 | Ile-Val |  | Stanton et al., 2015 |
|  |  |  |  | No association with cardiac markers | Lipshultz et al., 2013 |
|  | NR | NR | C282Y-Y | Significant correlation | Cascales et al., 2012 |
|  | NR | NR | H63D-H |  |  |
| HLA | NR | rs1800629 | TNF-a | Significant correlation | Todorova et al., 2017 |
|  | NR | rs2050190 | C6orf10 |  |  |
|  |  |  |  | Significant correlation | Visscher et al., 2013 |
|  |  |  |  | Presence of homozygote variant and heterozygote | Sachidanandam et al.,2012 |
|  | g.138780932T>C | rs17645700 | None | Significant correlation | Visscher et al., 2013 |
| HSD17B2 | g.82028304T>G | rs16956248 | None | SNP is associated with LVEF drop | Lubieniecka et al., 2013 |
|  | g.820829177T>A | rs13333826 | None |  |  |
|  | g.82054058C>T | rs7196087 | None |  |  |
|  | g.82079586C>T | rs2955159 | None |  |  |
|  | g.82072694G>A | rs2966245 | None |  |  |
| **Supplementary Table 1: Genetic polymorphisms associated with cardiotoxicity.** (continued) | | | | | |
| Gene | Nucleotide change | SNP rsID | Amino acid change | Association^2^ | Study  (Name, Year) |
| HSD17B4 | g.119502865G>T | rs257970 | None | SNP is associated with LVEF drop | Lubieniecka et al., 2013 |
|  | g.119502373G>A | rs2636968 | None |  |  |
| NCF4 | g.37256846GA>G | rs1883112 | None | No significant relationship | Visscher et al., 2012 |
|  |  |  |  |  | Hertz et al., 2016 |
|  |  |  |  |  | Reichwagen et al., 2015 |
|  |  |  |  | At risk genotype AA | Armenian et al., 2013 |
|  |  |  |  | Significant correlation | Rossi et al., 2009 |
|  |  |  |  | AA genotype is associated with chronic cardiotoxicity | Wojnowski et al., 2005 |
| NOS3 | g.150696111T>G | rs1799983 | p.Asp298Glu | Protective effect of the TT genotype on ejection fraction | Krajinovic et al., 2015 |
| NQO1 | g.69745145C>T | rs1800566 | Pro187Ser | At risk genotype CT/CC | Armenian et al., 2013 |
|  |  |  |  | Significant correlation | Blanco et al., 2008 |
|  |  | rs1043470 | T allele |  | Sági et al., 2018 |
| NR1I2 | g.119501039C>A | rs1523127 | None | No significant relationship | Hertz et al., 2016 |
|  | g.119530858G>A | rs3732357 | None |  |  |
|  | g.119499507T>C | rs1523130 | None |  |  |
| POR | g.75589903A>G | rs2868177 | None | SNP is associated with LVEF drop | Lubieniecka et al., 2013 |
|  | g.75606109G>A | rs13240755 | None |  |  |
|  | g.75607608C>T | rs4732513 | None |  |  |
|  | g.75601169G>A | rs6953065 | None |  |  |
| RAC2 | g.37236730T>A | rs13058338 | None | At risk genotype TA/AA | Armenian et al., 2013 |
|  |  |  |  |  |  |
| **Supplementary Table 1: Genetic polymorphisms associated with cardiotoxicity.** (continued) | | | | | |
| Gene | Nucleotide change | SNP rsID | Amino acid change | Association^2^ | Study  (Name, Year) |
|  |  |  |  | TA/AA genotypes are associated with cardiotoxicity | Wojnowski et al., 2005 |
|  |  |  |  | Significant correlation | Rossi et al., 2009 |
|  |  |  |  | No significant relationship | Reichwagen et al., 2015 |
|  |  |  |  |  | Hertz et al., 2016 |
|  |  |  |  |  | Visscher et al., 2012 |
| RARG | g.53605545G>A | rs2229774 | Ser4271Leu | OR 4.7 (2.7-8.3) | Aminkeng et al., 2015 |
| SERPINA6 | 93848406G>A | rs10144771 | None | Significant correlation | Visscher et al., 2015 |
| SLC10A2 | g.103714254G>A | rs9514091 | None | Protective variant | Visscher et al., 2012 |
|  | g.103723722G>A | rs7319981 | None | Significant correlation | Visscher et al., 2013 |
| SLC13A3 | 44693250A>G | rs2425886 | None | Significant correlation | Visscher et al., 2015 |
| SLC15A1 | 98153602G>C | rs8001466 | None | Significant correlation | Visscher et al., 2015 |
| SLC22A2 | g.160670282A>C | rs316019 | Ser270Ala | Noncausal relationship | Visscher et al., 2013 |
| SLC22A6 |  | rs6591722 |  | Significant correlation | Sági et al., 2018 |
| SLC22A7 | 43389166A>G | rs4149178 | None | Significant correlation | Visscher et al., 2015 |
| SLC22A16 | g.110778128T>C | rs714368 | His49Arg | Noncausal relationship | Hertz et al., 2016 |
|  | g.110777962A>G | rs6907567 | Asn104 |  |  |
|  | g.110763875A>G | rs723685 | Val252Ala |  |  |
|  | g.110760008A>G | rs12210538 | Met377Thr |  |  |
| SLC22A17 | 22884409G>A | rs4982753 | None | Significant correlation | Visscher et al., 2015 |
|  | 23814995A>T | rs11625724 | None |  |  |
|  | 23812237G>C | rs12882406 | None | Significant correlation after adjusting the effect of rs4982753 |  |
|  | 23816998T>C | rs12896494 | None |  |  |
| **Supplementary Table 1: Genetic polymorphisms associated with cardiotoxicity.** (continued) | | | | | |
| Gene | Nucleotide change | SNP rsID | Amino acid change | Association^2^ | Study  (Name, Year) |
| SLC28A1 | g.84909044T>C | rs2305364 | None | Significant correlation | Visscher et al., 2013 |
|  | g.84904404A>C | rs2290271 | None |  | Visscher et al., 2013 |
| SLC28A3 | g.86900926G>A | rs7853758 | Leu461 | Protective variant | Visscher et al., 2012 |
|  |  |  |  | Significant correlation | Visscher et al., 2013 |
|  |  |  |  | Noncausal relationship | Reichwagen et al., 2015 |
|  |  |  |  |  | Hertz et al., 2016 |
|  |  |  |  | Significant correlation | Sagi et al., 2018 |
|  | g.86946417A>C | rs4877847 | None | Protective variant | Visscher et al., 2012 |
|  |  |  |  | Significant correlation | Visscher et al., 2013 |
|  | g.86909550G>A | rs885004 | None |  | Visscher et al., 2013 |
| SLCO4C1 | g.101633540G>A | rs2600834 | None | Significant correlation | Visscher et al., 2015 |
| SLCO6A1 | g.101779552A>G | rs12658397 | None | Significant correlation | Visscher et al., 2015 |
| SOD2 | g.159981080G>A | rs7754103 | None | Significant correlation | Visscher et al., 2015 |
|  | g.160113872A>G | rs4880 | Val16Ala | Noncausal relationship between SNP and cardiac damage | Rajic et al., 2009 |
|  |  |  |  | At risk genotype GA/AA | Armenian et al., 2013 |
| SPG7 | g.89549357T>G | rs2019604 | None | Protective variant | Visscher et al., 2012 |
|  |  |  |  | Significant correlation | Visscher et al., 2013 |
| SULT2B1 | g.48588977C>A | rs10426377 | None | Significant correlation | Visscher et al., 2013 |
|  | 48589173A>G | rs10426628 |  |  | Visscher et al., 2015 |
| TLR | NR | NR | TLR2 | Significant correlation | Pop-Moldovan et al., 2017 |
|  | NR | NR | TLR4 |  |  |
| TP53 | g.757972G>C | rs1042522 | Pro72Arg | Noncausal relationship | Rossi et al., 2009 |
| **Supplementary Table 1: Genetic polymorphisms associated with cardiotoxicity.** (continued) | | | | | |
| Gene | Nucleotide change | SNP rsID | Amino acid change | Association^2^ | Study  (Name, Year) |
|  |  |  |  | Homozygote variant + heterozygote vs homozygote wt | Vivenza et al., 2013 |
| UGT1A6 | g.234601669T>G | rs6759892 | Ser7Ala | Risk variant | Visscher et al., 2012 |
|  |  |  |  | Significant correlation | Visscher et al.,2013 |
|  | g.234602277G>T | rs17863783 | Val209 |  | Visscher et al., 2013 |
|  | g.234593117G>T | rs4261716 | None |  | Visscher et al., 2013 |
| DSG2 | g.31521193T>G | rs191143292 | Val158Gly | Noncausal relationship | Wasielewski et al., 2014 |
| JUP | g.39913771C>T | rs143043662 | Val648Ile | Noncausal relationship | Wasielewski et al., 2014 |
| VCL | g.75873961C>T | rs150595117 | Ala990Val | Noncausal relationship | Wasielewski et al., 2014 |
| MYH7 | NR | NR | Asp(545,955)Asn | Noncausal relationship | Wasielewski et al., 2014 |
|  | NR | NR | Tyr1375 |  |  |
| TTN | NR | NR | Ser31346Leu | Noncausal relationship | Wasielewski et al., 2014 |
|  | g.179602871T>C | rs371552518 | Tyr4453Cys |  |  |
|  | NR | NR | Glu10855dup |  |  |
| TTNtv |  |  |  | Noncausal relationship | Garcia-Pavia et al., 2019 |
| DSP | NR | NR | Arg1425Lys | Noncausal relationship | Wasielewski et al., 2014 |
| PKP2 | g.32994058A>C | rs147240502 | Ile531Ser | Noncausal relationship | Wasielewski et al., 2014 |
| XDH | g.31460174G>A | rs4407290 | Val279 | Significant correlation | Visscher et al., 2015 |
|  | g.31460632A>G | rs2236168 | None |  |  |
|  |  |  |  |  |  |
